# Plasma Norepinephrine in Hypertensive Rats Reflects α_2_-Adrenoceptor Release Control Only When Re-Uptake is Inhibited

**DOI:** 10.3389/fneur.2012.00160

**Published:** 2012-11-08

**Authors:** Torill Berg, Sven Ivar Walaas, Bjørg Åse Roberg, Trang Thi Huynh, Jørgen Jensen

**Affiliations:** ^1^Department of Physiology, Institute of Basic Medical Sciences, University of OsloOslo, Norway; ^2^Department of Biochemistry, Institute of Basic Medical Sciences, University of OsloOslo, Norway; ^3^Department of Physical Performance, Norwegian School of Sport SciencesOslo, Norway

**Keywords:** α2-adrenoceptors, norepinephrine re-uptake transporter, hypertension, sympathetic nervous system activity, norepinephrine, epinephrine, catecholamine release, plasma catecholamine concentrations

## Abstract

α_2_-adrenoceptors (AR) lower central sympathetic output and peripheral catecholamine release, thereby protecting against sympathetic hyperactivity and hypertension. Norepinephrine re-uptake–transporter effectively (NET) removes norepinephrine from the synapse. Overflow to plasma will therefore not reflect release. Here we tested if inhibition of re-uptake allowed presynaptic α_2_AR release control to be reflected as differences in norepinephrine overflow in anesthetized hypertensive spontaneously hypertensive rats (SHR) and normotensive rats (WKY). We also tested if α_2_AR modulated the experiment-induced epinephrine secretion, and a phenylephrine-induced, α_1_-adrenergic vasoconstriction. Blood pressure was recorded through a femoral artery catheter, and cardiac output by ascending aorta flow. After pre-treatment with NET inhibitor (desipramine), and/or α_2_AR antagonist (yohimbine, L-659,066) or agonist (clonidine, ST-91), we injected phenylephrine. Arterial blood was sampled 15 min later. Plasma catecholamine concentrations were not influenced by phenylephrine, and therefore reflected effects of pre-treatment. Desipramine and α_2_AR antagonist separately had little effect on norepinephrine overflow. Combined, they increased norepinephrine overflow, particularly in SHR. Clonidine, but not ST-91, reduced, and pertussis toxin increased norepinephrine overflow in SHR and epinephrine secretion in both strains. L-659,066 + clonidine (central α_2_AR-stimulation) normalized the high blood pressure, heart rate, and vascular tension in SHR. α_2_AR antagonists reduced phenylephrine-induced vasoconstriction equally in WKY and SHR. Conclusions: α_2A_AR inhibition increased norepinephrine overflow only when re-uptake was blocked, and then with particular efficacy in SHR, possibly due to their high sympathetic tone. α_2A_AR inhibited epinephrine secretion, particularly in SHR. α_2A_AR supported α_1_AR-induced vasoconstriction equally in the two strains. α_2_AR malfunctions were therefore not detected in SHR under this basal condition.

## Introduction

α_2_-adrenoceptors (AR) are divided into three subtypes, i.e., α_2A_-, α_2B_-, and α_2C_AR. Through their ability to lower central sympathetic output and peripheral release of catecholamines from both sympathetic nerve terminals and the adrenal medulla, they play a significant role in preventing sympathetic hyperactivity (Starke, 2001). Deficiencies have been detected in the function of both central and peripheral α_2_AR in the spontaneously hypertensive rat (SHR) (Yamada et al., 1989; Remie et al., 1992; Reja et al., 2002; Zugck et al., 2003). Since deletion of the α_2A_AR–gene created a hypertensive mouse with high plasma norepinephrine levels (Makaritsis et al., 1999b), a failing α_2_AR control of catecholamine release may contribute to the elevated sympathetic activity (Judy et al., 1976) and high blood pressure (BP) in SHR. α_2_AR in vascular smooth muscle cells (VSMC) promote vasoconstriction, whereas endothelial α_2_AR stimulate nitric oxide synthesis and vasodilatation (Shafaroudi et al., 2005). Also these functions have been shown to be failing in SHR (Feres et al., 1998; Berg and Jensen, 2011). In addition, α_2_AR are involved in diabetes type II and behavior and cognitive disorders including attention-deficit hyperactivity (AD/HD) disorder (Hunt et al., 1995; Crassous et al., 2007; Fagerholm et al., 2011). Diabetes type II is comorbid with hypertension in the metabolic syndrome, and SHR is the most frequently used animal model for studying AD/HD (Arime et al., 2011).

The concentration of catecholamines in plasma is often used as an indication of adrenergic activity. Unlike that of adrenal epinephrine release, norepinephrine over flow to plasma will be influenced by re-uptake through the norepinephrine transporter (NET). The purpose of the present study was therefore to test if inhibition of re-uptake allowed differences in the plasma norepinephrine concentration to reflect the influence of presynaptic release control, here that of the α_2_-adrenceptor. We also tested if α_2_AR activity modulated adrenal epinephrine release and a provoked α_1_-adrenergic vascular tension-response, and if these functions differed in SHR and their normotensive controls (WKY). The results will demonstrate that presynaptic α_2_AR release inhibition was reflected as differences in the plasma norepinephrine concentration only when re-uptake was blocked, and then release inhibition was demonstrated to be far greater in SHR than in WKY. α_2_AR inhibited adrenal epinephrine secretion, also with greater efficacy in SHR, and stimulation of central α_2_AR lowered the elevated sympathetic tone and BP in this strain only.

## Materials and Methods

Ethical approval of the experiments was given by The Norwegian Animal Research Authority (NARA). All experiments were performed in accordance with the National Institutes of Health (NIH) Guide for the Care and Use of Laboratory Animals.

### Experimental procedure

Male, 12–14-weeks-old WKY (Wistar Kyoto, *n* = 79, 273 ± 4 g body weight) and SHR (Okamoto, SHR/NHsd strain, *n* = 81, 277 ± 3 g body weight) on 12/12 h light/dark cycles were allowed conventional rat chow diet (0.7% NaCl) and water *ad lib* until the time of the experiment. The rats were anesthetized with sodium pentobarbital (70–75 mg/kg, i.p.). As previously described (Berg et al., 2010), the rats were instrumented with a catheter in the femoral artery to monitor systolic (SBP) and diastolic (DBP) BP. Cardiac output (CO = minus coronary flow) and heart rate (HR) were measured with a 2SB perivascular flow probe on the ascending aorta and a T206 Ultrasonic Transit-Time Flowmeter (Transonic Systems Inc., Ithaca, NY, USA), entering the thoracic cavity through the third intercostal space. Mean arterial BP [MBP = (SBP + DBP)/3 + DBP] and total peripheral vascular resistance (TPVR = MBP/CO) were calculated. The thorax was closed with a suture, but the rats remained on a positive-pressure ventilator throughout the experiment, ventilated with air. Previous measurements of blood gas parameters demonstrated adequate ventilation in both strains (Berg, 2002, 2003). Body temperature was maintained at 37–38°C by external heating, guided by a thermo sensor inserted inguinally into the abdominal cavity. After completion of the surgery, the arterial catheter was flushed with 0.15 ml PBS (0.01 M Na-phosphate, 0.14 M NaCl, pH 7.4) containing 500 IU heparin/ml. Drugs were dissolved in PBS, and, unless otherwise indicated, administered as bolus injections (0.6–1 ml/kg) through a catheter in the femoral vein, flushed with 0.1 ml PBS.

### Experimental design

Rats were pre-treated with vehicle (PBS, −10 min), the peripherally restricted, non-selective α_2_AR antagonist L-659,066 (4.4 μmol/kg; Clineschmidt et al., 1988) or the non-selective, centrally active (Quaglia et al., 2011) α_2_AR antagonist yohimbine (5 μmol/kg, −10 min; Berg, 2003), alone or after prior administration of the NET inhibitor desipramine hydrochloride (44 μmol/kg, i.p., −5 h; Miralles et al., 2002). Rats were also pre-treated with the non-selective, centrally active α_2_AR agonist clonidine (151 nmol/kg, −15 min), alone or after prior administration of L-659,066 as above, or with the peripherally restricted α_2(non-A)_-selective agonist ST-91 (24 nmol/kg, −10 min; Takano et al., 1992). Since inhibitory G-protein (G_i_) represents a main signaling pathway for all α_2_AR, additional rats were injected with the G_i_-inhibitor *Bordetella pertussis* toxin (PTX, 15 μg/kg, i.p., −48 h; Berg et al., 2009), and pre-treated with PBS during the experiment. After pre-treatment, all rats were injected with phenylephrine (120 nmol/kg) to provoke an α_1_AR-mediated increase in TPVR. This concentration of phenylephrine was previously tested to activate a 3–4 times, but still sub-maximal, rise in TPVR. In addition, in a time-control group, phenylephrine was substituted with PBS. Control plasma was collected from anesthetized rats, which were not on respirator and not subjected to any surgery other than femoral artery catheterization.

### Measurement of plasma catecholamines

Arterial blood (1.5 ml) was collected 15 min after the injection of phenylephrine into tubes containing 40 μl 0.2 M glutathione and 0.2 M EGTA (4°C). Plasma was stored at −80°C until the catecholamine concentrations were determined using an HPLC-electrochemical detection method (Jensen et al., 2005).

### Statistical analyses

Results are presented as mean values ± SE mean. Each group comprised 6–8 rats. To include differences in baselines, the cardiovascular responses were expressed in % of baseline. The data were averaged every minutes throughout all experiments, every 5 s during the acute response to ST-91 and every 7th heart beat at the narrow TPVR-peak response to phenylephrine. Differences in the cardiovascular baselines, the response to pre-treatment, the TPVR-peak-response to phenylephrine and plasma catecholamine concentrations were evaluated by over-all tests (one-way ANOVA), followed by one- and/or two-sample Student’s *t*-tests. The clonidine-response curves were analyzed using Repeated Measures Analyses of Variance and Covariance, first as over-all tests within each strain, and subsequently for each group separately or between groups. Significant responses and group differences were then located using one- and two-sample Student’s *t*-tests, respectively, at specific times. In the presence of out-liers, the two-sample Student’s *t*-tests were substituted with non-parametric Kruskal–Wallis tests. At each step, testing proceeded only when the presence of significant responses and differences between groups was indicated. Bonferroni adjustment was performed for all tests, except for the catecholamine data, where *P* ≤ 0.05 was considered significant.

### Drugs

L-659,066 was a kind gift from Merck, Sharp, and Dohme Labs, Rahway, NJ, USA. ST-91 was from TOCRIS bioscience, Bristol, UK, and the remaining drugs from Sigma Chemical Co., St. Louis, MO, USA.

## Results

### The plasma concentrations of norepinephrine and epinephrine (Table 1)

**Table 1:** **The plasma concentration of norepinephrine and epinephrine**.

	WKY		SHR	
	Norepinephrine (nM)	Epinephrine (nM)	Norepinephrine (nM)	Epinephrine (nM)
Control plasma (no surgery)	0.6 ± 0.1	0.1 ± 0.1	0.8 ± 0.1	0.1 ± 0.1
Time-control (PBS + PBS)	0.9 ± 0.4	8.2 ± 1.3^†^	1.2 ± 0.3	11.4 ± 2.4^†^
PBS + phenylephrine	0.3 ± 0.1	4.3 ± 0.8	2.0 ± 0.3*	9.0 ± 1.9*
Clonidine + phenylephrine	0.4 ± 0.1	0.4 ± 0.1^‡^	0.5 ± 0.1^‡^	0.8 ± 0.2^‡^
ST-91 + phenylephrine	0.2 ± 0.1	3.6 ± 0.8	1.5 ± 0.6	7.7 ± 0.9
L-659,066 + phenylephrine	0.6 ± 0.1	7.3 ± 1.2^‡^	1.8 ± 0.5*	22.4 ± 5.5*^‡^
L-659,066 + clonidine + phenylephrine	0.8 ± 0.1	11.6 ± 4.9§	0.9 ± 0.1^‡^§║	5.6 ± 2.2§║
Yohimbine + phenylephrine	0.8 ± 0.8	7.2 ± 2.0	3.9 ± 0.7*^‡^	15.3 ± 2.4*
Desipramine + PBS + phenylephrine	0.8 ± 0.2	1.6 ± 0.5^‡^	1.7 ± 0.6	4.2 ± 1.6^‡^
Desipramine + L-659,066 + phenylephrine	1.9 ± 1.4	10.9 ± 7.2	30.2 ± 11.6*^‡^¶	16.3 ± 4.4¶
Desipramine + yohimbine + phenylephrine	2.2 ± 0.5^‡^¶	2.5 ± 1.5	17.6 ± 5.0*^‡^¶	11.6 ± 2.8*¶
PTX + phenylephrine	4.0 ± 1.7	49.2 ± 14.3^‡^	8.0 ± 1.1^‡^	50.5 ± 11.5^‡^

Differences were detected as indicated between corresponding SHR and WKY groups (* after SHR values), between control plasma and plasma from the time-control groups (†), between time-controls and the PBS + phenylephrine groups (significant differences not detected), between the PBS + phenylephrine controls and the corresponding experimental groups (‡), between groups pre-treated with L-659,066 + clonidine and clonidine (§) or L-659,066 alone (║), and between groups pre-treated with desipramine + PBS and desipramine + α_2_AR antagonist (L-659,066 or yohimbine) (¶). *, †, ‡, §, ║, ¶ *P* ≤ 0.05.

The concentration of norepinephrine in plasma collected at the end of the experiment in time-controls injected with PBS + PBS, did not differ from that in control plasma, collected from rats subjected to no other surgery than femoral artery catheterization. The concentration in the time-controls also did not differ from that in rats given PBS + phenylephrine. These results showed that the experiment itself and phenylephrine did not influence norepinephrine overflow to plasma.

The plasma concentration of norepinephrine in the phenylephrine-treated SHR controls was higher than that in WKY, and was reduced after pre-treatment with the centrally active, non-selective α_2_AR agonist clonidine. This reduction was in part eliminated by prior administration of the peripherally restricted, non-selective antagonist L-659,066. The peripherally restricted, α_2(non-A)_-selective agonist ST-91 did not influence norepinephrine overflow in either strain. α_2_AR antagonist (L-659,055 and yohimbine) and desipramine separately had no significant effect on the norepinephrine concentration, except for a minor increase after yohimbine in SHR. However, when combined, norepinephrine overflow increased, with particular efficacy in SHR. The G_i_-inhibitor PTX increased norepinephrine overflow in SHR, whereas the increase in WKY was not statistically significant.

The plasma concentration of epinephrine in the time-controls was higher than that in control plasma collected from rats not subjected to surgery, but was not different from that in the phenylephrine-treated controls. The plasma concentration of epinephrine in the phenylephrine-treated controls was higher in SHR than in WKY, but was in both strains reduced after pre-treatment with clonidine, and increased after L-659,066. The effect of L-659,066 was greater in SHR than in WKY. The reduction after clonidine was in both strains abolished by additional pre-treatment with L-659,066. The peripherally restricted α_2(non-A)_-selective agonist ST-91 did not influence the concentration of epinephrine. The secretion of epinephrine was reduced after desipramine in both strains, but α_2_AR antagonist still increased epinephrine secretion in desipramine-treated SHR. PTX greatly increased the plasma epinephrine concentration in both strains.

### The cardiovascular response to α_2_AR antagonists and agonists

**Figure 1:**
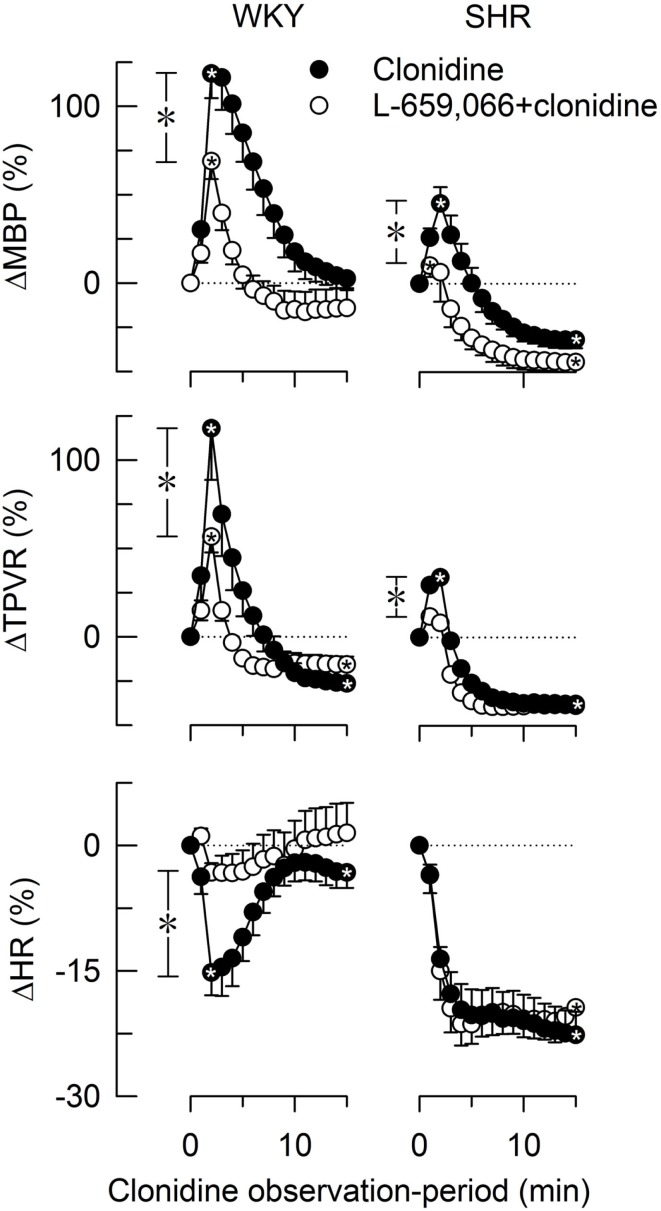
**The changes in MBP, TPVR, and HR in response to the centrally active, non-selective α_2_AR agonist clonidine, without or with prior administration of the peripherally restricted, non-selective α_2_AR antagonist L-659,066**. After curve evaluations, significant responses (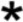
within symbols) and group differences at peak response (*in brackets left of curves) and at 15 min (*in brackets right of curves) were detected as indicated. Cardiovascular baselines prior to clonidine are shown in Table 2. 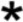,*P ≤ 0.025.

Baselines MBP, HR, and TPVR were higher in SHR than in WKY, whereas CO was less (Table 2). Clonidine-induced a transient increase in MBP and TPVR (Figure 1). ΔTPVR was less in SHR than in WKY. The peripherally restricted L-659,066 reduced this TPVR-response in WKY and eliminated the response in SHR. Clonidine subsequently reduced TPVR to below baseline in both strains, and L-659,066 had no effect on this response in either strain (Figure 1). The fall in tension following clonidine in the presence of L-659,066 was greater in SHR than in WKY, although TPVR remained higher in SHR (*P* < 0.001), but was not different from that in the WKY controls (*P* = NS; Table 2). The clonidine-induced reduction in TPVR was paralleled by hypotension in SHR only (Figure 1), eliminating the difference in MBP between the two strains (Table 2). Clonidine-induced an L-659,066-sensitive, transient bradycardia in WKY, but an L-659,066-insensitive, sustained bradycardia in SHR (Figure 1). The central effect of clonidine in SHR therefore eliminated the strain-related difference in baseline MBP, TPVR, and HR (Table 2).

**Table 2:** **Cardiovascular baselines prior to phenylephrine and, in parenthesis, the response to pre-treatment during the acute experiment**.

Pre-treatment	WKY				SHR			
	MBP mm Hg	HR beats/min	CO ml/min	TPVR mm Hg/ml/min	MBP mm Hg	HR beats/min	CO ml/min	TPVR mm Hg/ml/min
PBS	70 ± 4 (−3 ± 4)	328 ± 5 (−12 ± 8)	31 ± 3 (2 ± 2)	2.4 ± 0.2 (−0.3 ± 0.1)	91 ± 6* (−3 ± 6)	392 ± 11* (−16 ± 3)	19 ± 1* (−1 ± 1)	4.8 ± 0.5* (0.0 ± 0.2)
Clonidine	57 ± 5 (−2 ± 5)	310 ± 6 (−26 ± 7)	42 ± 2 (14 ± 1)^†^	1.4 ± 0.1^†^ (−0.8 ± 0.1)^†^	57 ± 3^†^ (−33 ± 7)*^†^	292 ± 6^†^ (−127 ± 17)*^†^	21 ± 1* (3 ± 1)*	2.7 ± 0.1*^†^ (−2.3 ± 0.3)*^†^
ST-91	75 ± 5 (−5 ± 3)	338 ± 7 (−20 ± 6)	32 ± 3 (2 ± 1)	2.6 ± 0.3 (−0.6 ± 0.2)	104 ± 9 (16 ± 7)	364 ± 11 (−36 ± 7)	21 ± 0 (1 ± 0)	5.0 ± 0.4 (0.6 ± 0.3)
L-659,066	52 ± 10 (−10 ± 2)	321 ± 14 (−6 ± 10)	24 ± 10 (1 ± 1)	2.1 ± 0.3 (−0.6 ± 0.2)	79 ± 9 (−7 ± 7)	437 ± 13* (4 ± 10)	21 ± 3 (1 ± 2)	4.2 ± 0.6 (−0.4 ± 0.2)
L-659,066 + clonidine	38 ± 2^†^^‡^ (−26 ± 8)^†^	303 ± 6 (−23 ± 11)§	34 ± 4 (5 ± 2)	1.2 ± 0.1^†^ (−1.2 ± 0.3)	43 ± 4^†^§ (−55 ± 7)^†^§	318 ± 12^†^§ (−89 ± 12)*^†^§	18 ± 3* (−3 ± 2)*	2.5 ± 0.2*^†^§ (−2.1 ± 0.2)*^†^§
Yohimbine	54 ± 5 (−12 ± 2)	277 ± 10^†^ (−38 ± 5)	30 ± 2 (1 ± 0)	1.8 ± 0.1 (−0.6 ± 0.1)	56 ± 4^†^ (−22 ± 5)	327 ± 7*^†^ (−42 ± 14)	15 ± 2* (−3 ± 2)	4.0 ± 0.3* (−0.7 ± 0.4)
PBS after desipramine	51 ± 5 (−4 ± 2)	329 ± 18 (−21 ± 13)	29 ± 3 (1 ± 1)	1.8 ± 0.3 (−0.2 ± 0.1)	64 ± 5 (−5 ± 2)	347 ± 10 (−24 ± 10)	16 ± 1* (−0 ± 1)	4.0 ± 0.3* (−0.2 ± 0.1)
L-659,066 after desipramine	41 ± 5^†^ (−12 ± 3)	314 ± 25 (−40 ± 0)^†^	21 ± 4 (−1 ± 2)	2.0 ± 0.2 (−0.5 ± 0.1)	88 ± 9* (9 ± 12)	486 ± 7^†^║ (32 ± 11)*^†^║	21 ± 2 (1 ± 1)	4.3 ± 0.3* (0.2 ± 0.4)
Yohimbine after desipramine	64 ± 4 (0 ± 1)	340 ± 9 (−18 ± 8)	36 ± 3 (3 ± 1)	1.8 ± 0.1 (−0.1 ± 0.0)	88 ± 7* (26 ± 2)*^†^║	459 ± 13*^†^║ (42 ± 9)*^†^║	22 ± 1*║ (3 ± 1)║	4.0 ± 0.2* (0.8 ± 0.1)*║
PBS after PTX	45 ± 4^†^ (−4 ± 2)	352 ± 8 (−9 ± 5)	37 ± 7 (−1 ± 3)	1.5 ± 0.3 (0.1 ± 0.2)	63 ± 7 (−4 ± 3)	429 ± 10* (−12 ± 3)	26 ± 5 (−2 ± 3)	3.0 ± 0.9 (0.0 ± 0.2)

Comparisons were made between the WKY and SHR controls (*), between the PBS-controls and the experimental groups (†), between groups pre-treated with L-659,066 + clonidine and clonidine (‡) or L-659066 alone (§), and between groups pre-treated with desipramine + PBS and desipramine + L-659,066/yohimbine (║). *P = 0.0125; †*P* = 0.0056; ‡, §, ║*P* = 0.025.

The peripheral, α_2(non-A)_-selective agonist ST-91 induced a transient increase in MBP and TPVR, followed by a reduction in CO, but had little effect on HR (not shown). After 10 min, MBP, HR, CO and TPVR did not differ from that in the controls (Table 2).

Baseline HR in both strains and MBP in SHR were lower after yohimbine (Table 2). After desipramine, L-659,066 reduced HR in WKY, but increased HR in SHR. Also yohimbine increased HR and, in addition, MBP and TPVR in desipramine-treated SHR. PTX lowered MBP in WKY only.

### The role of α_2_AR in modulating an α_1_AR-activated vasoconstriction

**Figure 2:**
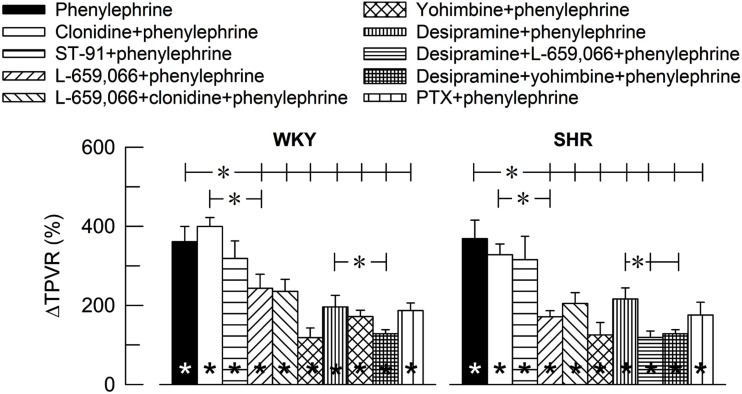
**The influence of α_2_AR on the TPVR-peak-response to the α_1_AR agonist phenylephrine**. The rats were pre-treated as indicated by symbol legends. TPVR prior to phenylephrine is shown in Table 2. 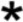
Within columns; significant responses. *In brackets; significant group differences. 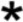
*P* ≤ 0.005; *P ≤ 0.0042.

The α_1_AR-selective agonist phenylephrine-induced a sharp and transient rise in TPVR (Figure 2) and MBP (ΔMBP = 118 ± 9 and 120 ± 14% in WKY and SHR, respectively) with negligible changes in HR (ΔHR = −7 ± 4%, *P* < 0.001, and −7 ± 1%, *P* = NS, respectively). The TPVR-response to phenylephrine was not different after pre-treatment with clonidine or ST-91, but was reduced after L-659,066, L-659,066 + clonidine, yohimbine, the NET inhibitor desipramine, and the G_i_-inhibitor PTX (Figure 2). Additional pre-treatment with L-659,066 or yohimbine in desipramine-treated rats, further reduced the TPVR-response to phenylephrine, except in desipramine + L-659,066-treated WKY. Significant strain-related differences in the TPVR-response to phenylephrine without or with pre-treatment were not detected.

## Discussion

The main finding in the present study was that peripheral α_2_AR antagonist increased norepinephrine overflow to plasma only when re-uptake was blocked, and then with a far greater efficacy in SHR than in WKY. α_2_AR inhibition of adrenal epinephrine release was also more pronounced in SHR. α_2A_AR appeared to be the main subtype responsible for peripheral catecholamine release inhibition. We also found that clonidine, through a central action, normalized the high MBP, HR, and TPVR in SHR, but strain-related differences in peripheral α_2_AR modulation of α_1_AR-mediated vasoconstriction were not detected.

Although all rats received phenylephrine between pre-treatment and the collection of blood, plasma catecholamine levels in the phenylephrine-control groups did not differ from that in time-controls injected with PBS instead of phenylephrine. From this observation, we concluded that phenylephrine itself did not influence the plasma catecholamine concentrations. Differences in the concentration of circulating catecholamines were therefore due to the treatment given prior to phenylephrine.

Norepinephrine is normally released from nerve terminal vesicles by exocytosis and removed from the synapse by re-uptake through NET. Vesicular release is Ca^2+^-dependent and under presynaptic control, and inhibited by presynaptic α_2_AR (Starke, 2001). Due to the efficacy of these two mechanisms, norepinephrine escape from the synapse into blood was low. α_2_AR antagonist or desipramine alone had no or only little effect on norepinephrine overflow in either strain, indicating that inhibition of one mechanism was compensated by the other. However, α_2_AR antagonist and desipramine combined increased the plasma norepinephrine concentration, and with particular efficacy in SHR.

Unlike that observed for norepinephrine, the plasma concentration of epinephrine in rats exposed to a full experiment was higher than that in control rats, not exposed to artificial ventilation or surgery other than arterial catheterization. This epinephrine secretion was therefore caused by the experiment itself, probably due to surgical stress and activation of the sympatho-adrenal axis. NET did not influence adrenal epinephrine overflow, since the epinephrine concentration in plasma was not increased by tyramine (Berg et al., 2010), which activates reverse transport through NET. The elevated plasma norepinephrine concentration after α_2_AR antagonist in desipramine-treated rats was therefore not paralleled by an increase in circulating epinephrine. In fact, desipramine reduced the secretion of epinephrine in both strains, most likely through its action on central pathways (Eisenhofer et al., 1991).

In agreement with that clonidine lowered central sympathetic output and BP in conscious rats by stimulating central α_2A_AR (MacMillan et al., 1996; Makaritsis et al., 1999a; Philipp et al., 2002), clonidine in the presence of the peripherally restricted L-659,066, *i.e.*, through a central action, normalized the high MBP, HR, and TPVR baselines in SHR. Clonidine in the presence of L-659,066 also lowered norepinephrine overflow to plasma in SHR only. The central sympathetic tone was therefore clearly elevated in SHR, and could be lowered by clonidine. This observation showed that a high sympathetic tone played an important role in sustaining the hypertension in SHR, possibly due to an insufficient α_2_AR control of central sympathetic output. However, the α_2_AR were present and functional since they did respond to agonist.

Peripheral α_2_AR actively inhibited norepinephrine release in SHR, demonstrated by the increase in overflow to plasma after the peripheral antagonist L-659,066, and also yohimbine, when re-uptake was blocked with desipramine. The overflow was by far greater in SHR than in WKY. This observation may be explained by the elevated sympathetic tone in this strain, resulting in a higher peripheral, vesicular release, with subsequent, enhanced auto-activation of the presynaptic, release-inhibiting α_2_AR. Representing the last line of defense against sympathetic hyperactivity, it is also possible that the α_2_AR were in fact up-regulated in SHR, in order to prevent excessive release due to the elevated sympathetic tone in this strain.

Peripherally restricted, subtype-selective α_2_AR agonists or antagonists are not available. However, clonidine reduced norepinephrine overflow in SHR, and the secretion of epinephrine in both strains. The reduction in norepinephrine overflow was in part reversed and that of epinephrine fully reversed by the peripherally restricted antagonist L-659,066, demonstrating involvement of peripheral α_2_AR. Similar reductions were not observed after the peripheral, α_2(non-A)_-selective agonist ST-91. We therefore deduced that the α_2A_AR was the main subtype responsible for peripheral inhibition of norepinephrine and epinephrine release in both WKY and SHR. This conclusion was compatible with previous studies on genetically modified mice, where α_2A_AR inhibited release at high stimulation frequencies, whereas inhibition of α_2C_AR hampered release at lower stimulation frequencies (Hein et al., 1999). Since sympathetic output was clearly enhanced in SHR, the basal sympathetic tone in this strain may resemble the experimental higher frequencies. Furthermore, inhibition of epinephrine release from the adrenal medulla involved the α_2A_-subtype in rat and man (Lymperopoulos et al., 2007), although the α_2C_-subtype in the mouse (Brede et al., 2003; Moura et al., 2006). α_2A_AR catecholamine release inhibition apparently signaled through G_i_, since PTX increased the plasma concentration of norepinephrine in SHR and greatly that of epinephrine in both strains. The more prominent effect of PTX on the plasma concentration of epinephrine compared to that of norepinephrine was likely to be explained by norepinephrine re-uptake through NET.

Catecholamine-induced vasoconstriction is mediated not only through the α_1_AR-phospholipase C pathway, but also by α_2_AR. VSMC α_2_AR may signal through G_i_, and, in that manner, oppose the vasodilatory effect of the adenylyl cyclase – cyclic AMP pathway, activated by VSMC βAR and stimulatory G-protein. A shift in the VSMC α_2_AR – βAR balance may therefore explain why α_2_AR antagonist and PTX reduced the phenylephrine-induced, α_1_AR-mediated vasoconstriction. The VSMC α_2_AR tone appeared maximally stimulated, since it could not be further stimulated by clonidine or ST-91. Unlike the effect of α_2_AR antagonist on norepinephrine overflow, α_2_AR antagonist modulated the TPVR-response to phenylephrine also in the absence of NET inhibitor. This was as expected, since activation of postsynaptic α_2_AR takes place within the synapse as part of the physiological response, and likely to be less sensitive to NET re-uptake than overflow to plasma. Still, desipramine alone reduced the TPVR-response to phenylephrine. This may be due to the central sympatholytic action of desipramine, or an increased βAR vasodilatation following the increased concentration of norepinephrine within the synapse when re-uptake was blocked. When the TPVR-response to phenylephrine was expressed in integers of baseline, strain-related differences in the α_2_AR modulation were not detected, even though malfunctions have been reported for VSMC α_2_AR in SHR (Feres et al., 1998). The smaller, initial clonidine-induced vasoconstriction in SHR compared to WKY, was most likely explained by an overlap from the subsequent reduced sympathetic tone due to activation of central α_2_AR in this strain.

The present experiments were performed on pentobarbiturate-anesthetized rats on a positive-pressure ventilator. Due to the anesthesia, the large increase in MBP induced by phenylephrine had hardly any effect on HR, similar to that previously observed during an acute fall in MBP activated by bradykinin (Bjørnstad-Østensen and Berg, 1994). The observed cardiovascular responses were therefore without interference from baroreflex activation. However, the positive-pressure ventilation hampered thoracic venous return to the right atrium, and, hence, lowered stroke volume, CO, and BP, but had little effect on baseline TPVR. This effect was more prominent in SHR than in WKY. However, this influence did not differ between the groups within each strain, and therefore was not likely to have an impact on the response to the pharmacological interventions or on the conclusions made there from.

The 5-HT_1A_-agonistic effect of yohimbine may through presynaptic intervention inhibit norepinephrine release (Moran et al., 1998; Quaglia et al., 2011). This activity did not seem to play a prominent role in its effect on release, since yohimbine alone in SHR, and in the presence of desipramine in both strains, increased norepinephrine overflow to plasma. Activation of a central 5-HT_1A_-component may explain why yohimbine lowered baseline HR (Villalon and Centurion, 2007). However, both α_2_AR antagonists increased HR and to some extent also TPVR in the desipramine-treated SHR. This increase was likely to result from a high norepinephrine synaptic concentration in these two groups.

## Conclusion and Implications

α_2_AR antagonist did not increase norepinephrine overflow to plasma unless re-uptake through NET was blocked. Thus, unless NET re-uptake was prevented, the norepinephrine concentration in plasma was not a good indicator of sympathetic nerve activity, and not suited for studying presynaptic norepinephrine release control. The present experiments provided the conditions under which the plasma concentrations will reflect differences in catecholamine release so that mechanisms influencing release can be studied in whole animal experiments.

α_2_AR malfunctions in the peripheral control of catecholamine release or the support of α_1_AR-mediated vasoconstriction were not detected in SHR. This may be different when the sympathetic nervous system is activated, since the inhibitory effect of PTX on vasoconstriction evoked by tyramine-stimulated norepinephrine release was less in SHR than in WKY (Berg et al., 2009), whereas a strain-related difference was not seen for the effect of PTX on the present phenylephrine-induced vasoconstriction.

α_2A_ appeared to be the main α_2_AR subtype responsible for peripheral inhibition of norepinephrine in SHR and epinephrine release in both strains, both signaling through PTX-sensitive G_i_. The high central sympathetic tone and cardiovascular baselines in SHR were normalized by stimulation of central α_2_AR. This elevated sympathetic tone may be the reason why the effect of α_2_AR antagonist on catecholamine release was greater in SHR than in WKY. The high norepinephrine concentration in plasma after α_2_AR antagonist in the presence of NET inhibitor may be utilized for diagnostic purposes to identify patients with hypertension due to sympathetic hyperactivity. Also a clonidine-induced, sustained bradycardia in the presence of peripherally restricted α_2_AR antagonist, appeared to be specifically related to the sympathetic hyperactivity in SHR. Such diagnostics tools are needed in order to select those patients who will benefit from newly developed therapies targeting the autonomic imbalance in hypertension, such as implanted barostimulator and renal nerve ablation.

## Conflict of Interest Statement

The authors declare that the research was conducted in the absence of any commercial or financial relationships that could be construed as a potential conflict of interest.
